# Supplementary material for: An open-source computational tool for measuring bacterial biofilm morphology and growth kinetics upon one-sided exposure to an antimicrobial source
Source: Sci Rep. 2022 Sep 27;12:16125. doi: 10.1038/s41598-022-20275-8 (PMC9515175; doi:10.1038/s41598-022-20275-8)
Supplement: Supplementary file 1 — Supplementary Information. [file 41598_2022_20275_MOESM1_ESM.pdf]

An open-source computational tool for measuring bacterial biofilm morphology and growth kinetics upon one-sided exposure to an antimicrobial source

1. Installation

Our program is compatible with Python 3 on Windows/Linux.

1. Install Python 3:
   https://www.python.org/download/releases/3.0/
2. Download freely available code from:
   https://github.com/cohenoa/An-Open-Source-Computational-Tool-for-Measuring-Bacterial-Biofilm-Morphology-and-Growth-Kinetics
3. Unzip the downloaded file.
4. Rename the unzipped folder (optional): <CODE_FOLDER>, e.g., “C:\BiofilmSoftware\”
5. Open the command line (Windows command: `cmd`)
6. Go to the folder:

```
cd C:\BiofilmSoftware\
```

7. Run the following command (installation of necessary software components):

```
pip install -r requirements.txt
```

2. Execution

2.1 Input and Output folders preparation

Before executing the program, prepare an input folder which will contain your biofilm images and an additional folder which will contain the center coordinates of the input images. For more detail, you can refer to the input folder that was used for our experiments (“Dataset”).

Additionally, create an output folder, into which all figures generated by the software will be automatically saved. This step is optional, as the program will automatically

create a folder in the destination specified in the execution command if it does not exist.

2.2 Execution command

```
python main.py -i <INPUT_FOLDER> -o <OUTPUT_FOLDER>
```

For example, if the input folder is “C:\UserInput\” and the output folder is “C:\Output\”, the command is:

```
python main.py -i C:\UserInput\ -o C:\Output\
```

2.3 Working example

```
C:\>cd C:\BiofilmSoftware

C:\BiofilmSoftware>pip install -r Requirements.txt

C:\BiofilmSoftware>python main.py -i C:\UserInput\ -o C:\Output\
```

The output folder will contain the resulting figures, numbered according to their references in the paper:

C:\Output

Name	Type	Size
Figure_1a.png	PNG File	294 KB
Figure_1b.png	PNG File	45 KB
Figure_2a.png	PNG File	112 KB
Figure_2b.png	PNG File	62 KB
Figure_3a.png	PNG File	89 KB
Figure_3b.png	PNG File	54 KB
Figure_4.png	PNG File	39 KB
Figure_5.png	PNG File	239 KB
Figure_6.png	PNG File	54 KB
